# Supplementary material for: Enhancer RNA Expression in Response to Glucocorticoid Treatment in Murine Macrophages
Source: Cells. 2021 Dec 23;11(1):28. doi: 10.3390/cells11010028 (PMC8744892; doi:10.3390/cells11010028)
Supplement: Supplementary file 1 [file cells-11-00028-s001.zip › cells-1462659-supplementary proof done/Supplementary Materials_rev proof done.pdf]

Supplementary Materials

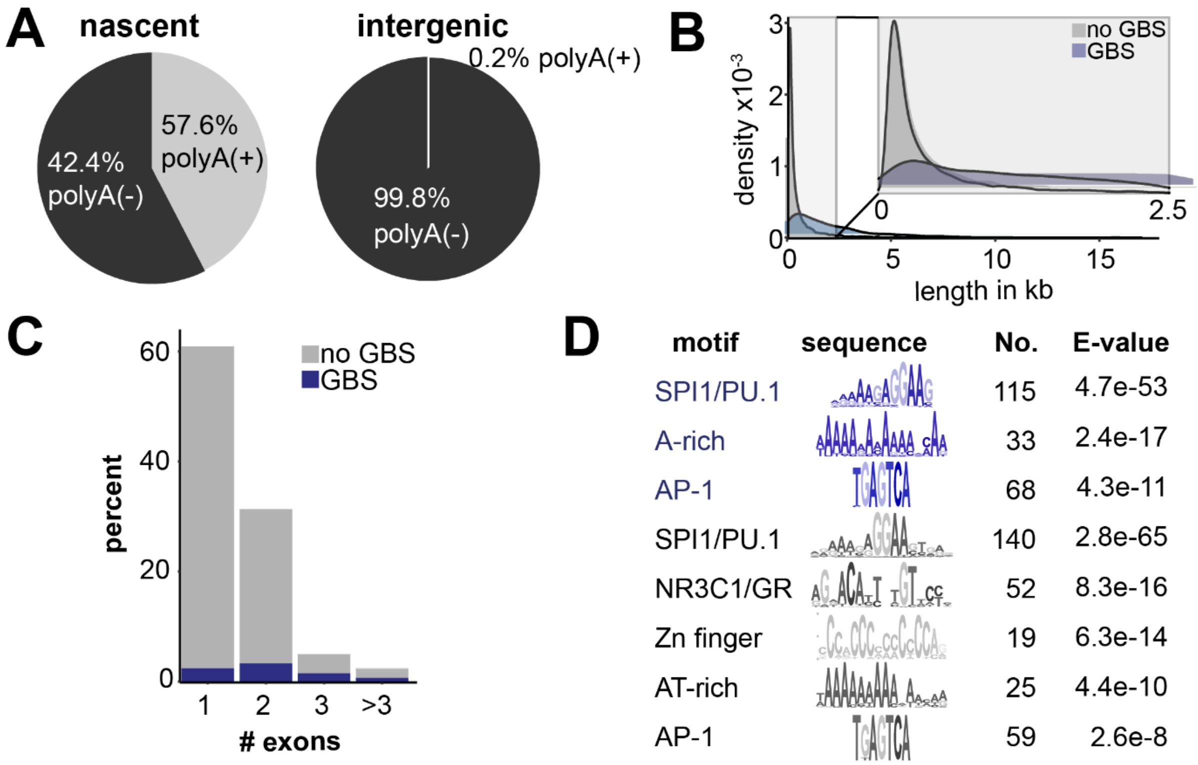

Figure S1. Characterization of nascent transcripts from GR binding sites in murine macrophages after LPS +Dex stimulation. This figure relates to Figure 1. (A) Fraction of nascent RNAs also identified by PolyA-selected (polyA(+)) RNA-seq. Left: all expressed nascent transcripts. Right: intergenic nascent transcripts (iRNAs) as identified in Figure 1A (left). (B) Density plot of the iRNA length distribution comparing iRNAs overlapping GBSs and iRNAs not overlapping GBSs. A magnification of the 0-2.5 kb length range is shown in the grey square. (C) Percent of iRNAs with 1, 2, 3 or more predicted exons stratified by their overlap with or without any iGBS. Bars reflect the percentage of all iRNAs. (D) Motifs enriched at GBS with eRNA (blue) and GBS without detectable eRNA production (gray). The number of sequences with motif (No.) and the MEME E-value are given.

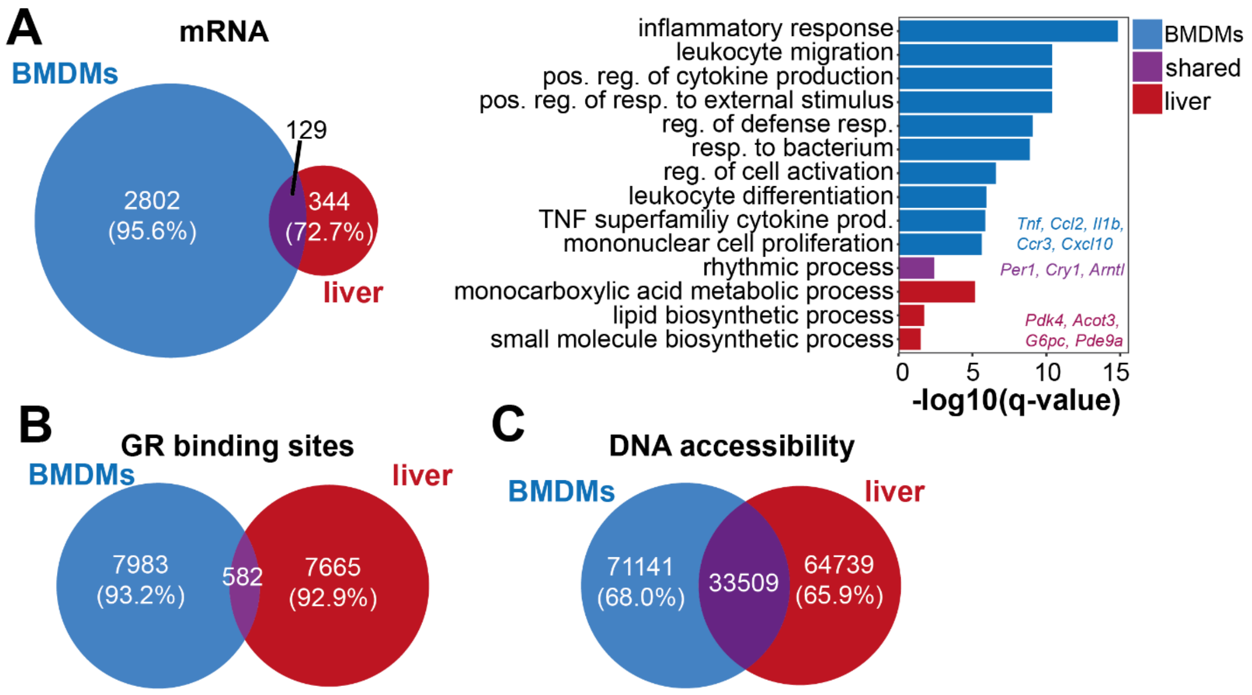

Figure S2. Tissue-specific glucocorticoid actions. This figure relates to Figure 2. (A) Differentially expressed genes in response to GCs in macrophages (LPS+Dex treatment, BMDMs) and liver tissue (ZT12 vs ZT0). Left: The overlap as Venn diagram. Percentage reflect the fraction of tissue-specifically regulated genes. Right: Gene Ontology over-representation analysis for biological processes of genes stratified by tissue expression (top 10 non-redundant biological processes with q-value <0.05, examples are listed). pos. =positive, reg. =regulation, resp. =response. (B) Venn diagram showing the overlap in GR binding sites (ChIP-seq, n=2). (C) Overlap of DNA accessible sites in LPS plus Dex-treated BMDMs (ATAC-seq, n=4) and liver at ZT14 (DHS, n=1). (B+C) Percentage reflect the fraction of tissue-specific sites.

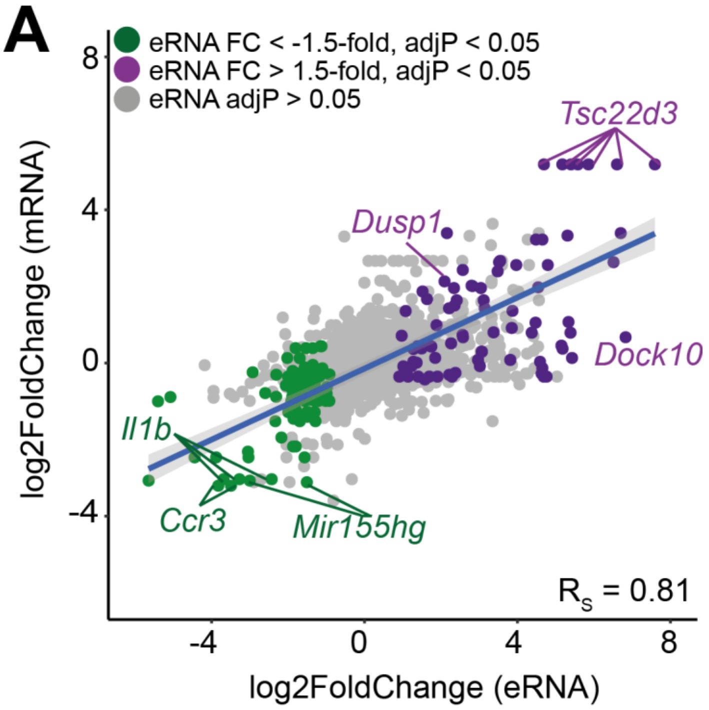

Figure S3. Correlation of mRNA and eRNA transcription. This figure relates to Figure 3. (A) Scatter plot of the log2 fold-change in eRNA and associated mRNA expression in macrophages after LPS+Dex treatment when compared

to LPS stimulated cells as determined by 4sU-seq. A linear regression for those eGBSs with significant (Benjamini-Hochberg adjusted p-value < 0.05) changed expression upon Dex treatment was performed (blue, grey shadow: 95% confidence interval). The Spearman correlation coefficient (RS) is given. n=3

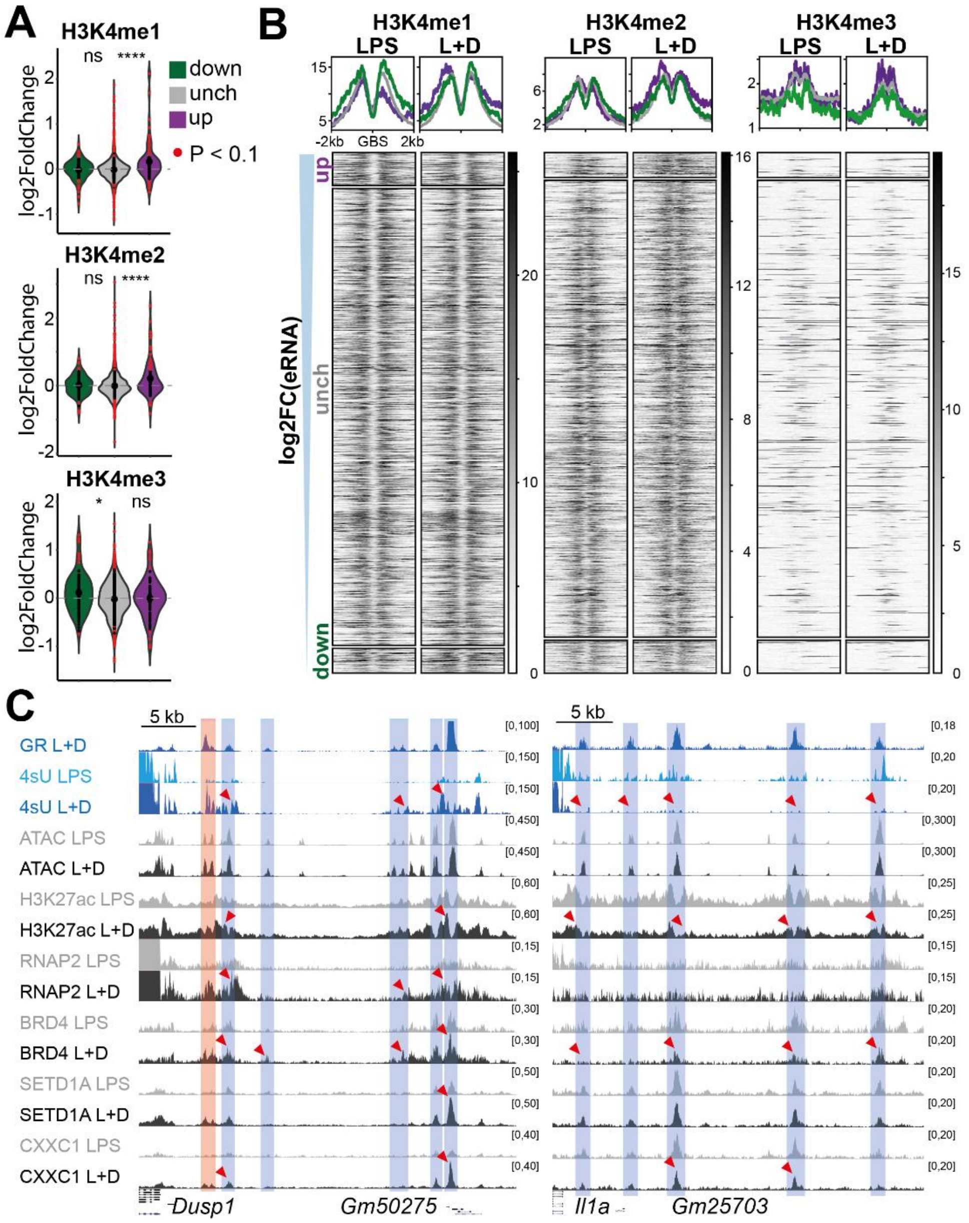

Figure S4. eRNA expression from GBSs correlates with BRD4 and H3K27ac. This figure relates to Figure 4. (A) Distribution of log2 fold-changes in H3K4me1/me2 or me3 between LPS and LPS+Dex-treated macrophages. eGBSs are grouped by significantly up-, down-regulated or non-significantly changed (adjP > 0.05, unch, see Figure 3A). Each dot represents one enhancer. Red denotes significance (p<0.1) (ChIP-seq, Wilcoxon-Mann-Whitney test, ****p<0.00001, *p<0.01, ns- non-significant) (B) Mean heatmaps of ChIP-seq signal against H3K4me1/me2 or me3 in LPS or LPS+Dex (L+D) stimulated macrophages grouped by eGBSs with induced (up, top), repressed (down, bottom) or non-significantly (adjP > 0.05) changed eRNA expression (unch, middle). eGBSs are sorted in descending order of the log2 fold-change. The coverage plot summarizes the median density of ChIP-seq signal within each eGBS group (n=2). Colors as in A. (D) Genome browser tracks of an eGBS with induced eRNA (*Dusp1*, left) or repressed eRNA expression (*Il1a*, right) upon Dex stimulation in LPS treated BMDMs. Tracks are means of biological replicates. Blue shades indicate eGBSs. Red shades mark eGBSs with alterations of all displayed features. Red arrow heads point towards changes upon Dex treatment. (n=4 for ATAC-seq, n=3 for 4sU-seq; n=2 for H3K27ac, RNAP2, BRD4, SETD1A, CXXC1 and GR ChIP-seq)

Supplementary Tables

Table S1: NGS data sets used in this study including accession numbers, treatment conditions, references, mapping statistics and normalization factors. This table relates to the Data Sources part of the method section.

Table S2: Overrepresentation analysis for biological processes of tissue-specifically regulated glucocorticoid response genes (BMDMs and liver). This table relates to figure S2A.

Table S3: GR binding sites with eRNA expression in BMDMs upon Dex and LPS stimulation and/or liver tissue at the peak of endogenous glucocorticoid levels. This table relates to figures 1 and 2.

Table S4: Overrepresentation analysis for biological processes of tissue-specifically expressed eGBSs. This table relates to figure 2B.

Table S5: Overrepresentation analysis for biological processes of genes associated with differentially expressed eGBSs in LPS-treated BMDMs upon Dex stimulation. This table relates to figure 3B.
